# Microbial dynamics of acute pancreatitis: integrating culture, sequencing, and bile impact on bacterial populations and gaseous metabolites

**DOI:** 10.3389/fmicb.2025.1544124

**Published:** 2025-02-12

**Authors:** Agnieszka Chmielarczyk, Edyta Golińska, Anna Tomusiak-Plebanek, Natalia Żeber-Lubecka, Maria Kulecka, Antoni Szczepanik, Katarzyna Jedlińska, Krzysztof Mech, Konrad Szaciłowski, Agata Kuziak, Agata Pietrzyk, Magdalena Strus

**Affiliations:** ^1^Department of Microbiology, Faculty of Medicine, Jagiellonian University Medical College, Kraków, Poland; ^2^Department of Gastroenterology, Hepatology and Clinical Oncology, Center of Postgraduate Medical Education, Warsaw, Poland; ^3^Department of Genetics, Maria Sklodowska-Curie National Research Institute of Oncology, Warsaw, Poland; ^4^Clinical Department of General Surgery and Oncology, Narutowicz City Speciality Hospital at Krakow, Krakow, Poland; ^5^Department of Analytical Chemistry and Biochemistry, Faculty of Materials Science and Ceramics, AGH University of Science and Technology of Krakow, Krakow, Poland; ^6^Academic Center for Materials and Nanotechnology, AGH University of Krakow, Krakow, Poland

**Keywords:** acute pancreatitis, microbiome, microbiota, bile, NGS, gaseous metabolites, *Fusobacterium nucleatum*, *Escherichia coli*

## Abstract

**Background:**

Our study examined the composition of the intestinal microflora in a hospitalized patient with AP symptoms treated several months earlier for diverticulitis. The therapeutic intervention necessitated Hartmann's procedure, culminating in colostomy creation.

**Aims:**

Employing a thorough microbiological analysis we attempted to demonstrate whether the microflora isolated from the peripancreatic fluid exhibited a stronger correlation with the contents of the stoma or with the rectal swab. Additionally, we sought to determine the association between later onset of AP and diverticulitis.

**Methods:**

Following clinical materials from the patient in the initial phase of AP were collected: rectal swab, colostomy bag contents (in the publication referred to as stoma content/stool) and peripancreatic fluid. Microbiological analysis was performed, including classic culture methodology, NGS techniques, and genotyping methodologies. Furthermore, the effect of bile on the shift in the population of selected bacterial species was examined.

**Results:**

The NGS technique confirmed greater consistency in bacteria percentage (phyla/family) between stoma content and peripancreatic fluid. In both samples, a clear dominance of the Proteobacteria phyla (over 75%) and the Enterobacteriaceae family was demonstrated. Moreover, NGS verified the presence of the Fusobacteriota phylum and Fusobacteriaceae family only in rectal swabs, which may indicate a link between this type of bacteria and the etiology of diverticulitis. We observed that Escherichia coli 33 isolated from stool exhibited active gaseous metabolite production (mainly hydrogen).

**Conclusions:**

The abundant production of hydrogen may substantially impact enzymatic processes, inducing specific alterations in disulfide bonds and trypsin inactivation. Our investigation alludes to the conceivable active involvement of bile in effecting qualitative and quantitative modifications in the peripancreatic microbiota composition, establishing a correlation between released bile and bacterial generation of gaseous metabolites.

## 1 Introduction

According to the most recent epidemiological data and literature, a marked surge in the incidence of acute pancreatitis (AP) among middle-aged and elderly populations in developed nations has become apparent (Pendharkar et al., 2017). Notably, regions with a high AP prevalence (exceeding 30 cases per 100,000) include the United States and Canada. In contrast, European countries exhibit AP prevalence ranging from 29 to 12 cases per 100,000 inhabitants annually (Petrov and Yadav, 2019). Until now, gastroenterologists, surgeons, and epidemiologists have subscribed to the prevailing belief that prominent predisposing factors for AP encompass gallbladder and bile duct dysfunction, alcohol misuse, immunosuppressive agents, a stress-laden lifestyle, improper dietary practices, escalating obesity rates, abdominal traumas, and parathyroid gland hyperfunction (Wang et al., 2013). Nevertheless, gallstones persist as the foremost etiological factor in AP (constituting 45% of the cases), closely followed by alcohol misuse (20% of the total number of cases) (Boxhoorn et al., 2020; Hu et al., 2021).

Contemporary investigations by specific research cohorts have recently postulated an association between AP, ongoing intestinal inflammation, and surgical interventions performed on the intestines, such as those involving a stoma creation (Jessen et al., 2021).

AP manifests through both localized and systemic inflammatory reactions, classified into distinct categories as mild AP (MAP), moderately severe AP (MSAP), and severe AP (SAP), as per the 2012 revised Atlanta Classification proposed by Banks et al. (2012). The overall mortality rate associated with AP is generally reported at 1%−3%, yet in cases where SAP is present, the mortality rate can escalate markedly, reaching up to 30% (Hu et al., 2021; Petrov et al., 2010).

Previously established knowledge underscores the negligible involvement of the human digestive tract microflora in MAP and MSAP onset. This assertion rests upon the infrequency of microbiological analyses conducted on clinical samples derived from the peripancreatic space in individuals presenting with the initial stages of pancreatitis. Moreover, when such investigations are undertaken, microbial cultures are either negative or contaminated with many species characteristic of the gut microbiota. For MAP patients, the condition typically follows a self-limiting course with conservative management. In instances of MSAP, neither percutaneous nor surgical interventions are typically undertaken unless there are discernible clinical symptoms suggestive of potential infection. In cases of SAP, bacteria isolated from the peripancreatic space aggressively contribute to ongoing inflammatory processes, swift patient deterioration, tissue necrosis, sepsis, multiorgan failure, and fatality (Patel et al., 2021; Boumitri et al., 2017). Notably, Gram-negative rods (*Enterobacterales*), alongside Gram-positive cocci (*Enterococcus faecalis* and *Enterococcus faecium*), are frequently isolated from necrotizing pancreatitis in SAP patients. In this context, the predominance of Gram-negative rods is particularly implicated as a determinant leading to patient fatality (Patel et al., 2021; Zhu et al., 2019).

Currently, utilizing NGS, the capacity to scrutinize alterations in the microbiome across distinct stages of pancreatitis (encompassing MAP, MSAP, and SAP) has significantly increased. The applicability of NGS analyses extends comprehensively to all gastrointestinal tract samples, even in the aftermath of antibiotic administration, owing to its exclusive reliance on bacterial DNA originating from viable and non-viable bacterial entities.

In our investigative pursuits, we meticulously examined the compositional dynamics of the intestinal microbiota in a hospitalized patient presenting with AP symptoms. This particular patient, previously treated for diverticulitis a few months prior, was slated for the removal of a colostomy following the Hartmann procedure.

Facilitated by an exhaustive microbiological analysis, incorporating classical culture methodology, NGS techniques, and genotyping methodologies, we attempted to elucidate whether the microbiota isolated from the peripancreatic space demonstrated a stronger correlation with the stoma contents or the rectal microbiota. Through comparative assessment of these intestinal microbiotas, our objective was to discern potential associations between subsequent occurrences of acute pancreatitis and a medical history featuring diverticulitis or colostomy procedures.

Furthermore, we sought to investigate the impact of freshly secreted bile on the qualitative and quantitative composition of gas metabolites produced by a specific strain of *Escherichia coli*, thus expanding our understanding of the intricate interplay between bile constituents and the microbiota in the context of AP.

## 2 Materials and methods

The microbiological analyses encompassed the procurement of samples (rectal swab, stoma content/stool, and peripancreatic fluid) from a 39-year-old individual presenting with MSAP. The patient's admission to the Surgery Department of the University Hospital at Jagiellonian University Medical College occurred in February 2020, prompted by abdominal pain indicative of large bowel obstruction resulting from acute diverticulitis. The therapeutic intervention necessitated Hartmann's procedure, culminating in the creation of colostomy. After hospital discharge (the patient's condition was satisfactory), a scheduled date for restoring digestive tract continuity was established.

Several days before the planned restorative procedure on June 22, 2020, the patient had experienced a recurrence of severe abdominal pain accompanied by sweating, obstruction, and no colostomy bag contents. AP was confirmed upon conducting blood tests, abdominal ultrasound, and abdominal computed tomography. Imaging studies further identified acute-phase peripancreatic fluid collection. Given the patient's deteriorating general condition, a transfer to the intensive care unit (ICU) ensued for intensified therapeutic measures. In the ICU, a comprehensive array of clinical specimens was systematically collected for meticulous microbiological scrutiny, including blood, urine, rectal swabs, stoma content/stool, and peripancreatic fluid (obtained percutaneously).

Following the initial blood culture, wherein solely *Staphylococcus epidermidis* was detected (with suspicion of sample contamination), the persistent elevation in body temperature, reaching up to 39 degrees Celsius, prompted the initiation of broad-spectrum empirical antibiotic therapy. The regimen included Ciprofloxacin (three doses of 400 mg), Metronidazole (three doses of 500 mg), Xifaxan (three doses of 200 mg), and Diflucan (two doses of 200 mg). Subsequent blood culture analysis revealed the isolation of *Staphylococcus capitis* methicillin-resistant coagulase negative staphylococci (MSCNS), exhibiting sensitivity to isoxazolyl penicillins, combinations of penicillins with beta-lactamase inhibitors, cephalosporins (excluding ceftazidime, cefixime, ceftibuten), and carbapenems. Consequently, the antibiotic protocol was modified to incorporate Meropenem (three doses of 2 g), Metronidazole (three doses of 500 mg), Xifaxan (three doses of 200 mg), and Diflucan (two doses of 200 mg).

Throughout this therapeutic course, the patient received oral and parenteral nutrition with commendable tolerance, as evidenced by colostomy discharge. Continuous intravenous patient-controlled intraocular pressure (IOP) anesthesia was maintained. Noteworthy improvements, characterized by a reduction in inflammatory parameters and an enhanced health condition, were discerned on July 6, 2020. Subsequently, the patient (in a favorable condition) was transferred back to the Surgery Department of the University Hospital at Jagiellonian University Medical College in Kraków for ongoing therapeutic interventions.

Microbiological sample collection was conducted following the Declaration of Helsinki and approved by the Bioethics Committee of the Jagiellonian University (opinion number KBET 1072.6120.279).

### 2.1 The qualitative and quantitative classical culture method

The specimens acquired from the patient (rectal swab, stoma content/stool, and peripancreatic fluid) were meticulously processed during the onset of exacerbated MSAP clinical manifestation. The Department of Microbiology at Jagiellonian University Medical College conducted qualitative and quantitative microbiological cultures. Post-collection, the materials were transported in sterile containers on dry ice within 2 h. Subsequently, the samples were suspended in Schaedler's liquid medium (Becton, Dickinson and Company, Sparks, MD, USA), precisely weighed, and finely ground using sterile mortars and glass beads. Decimal dilution methodology was employed, and the diluted samples were plated on appropriate agar media.

Distinct agar media were utilized for specific bacterial groups:

- McConkey agar (Oxoid Ltd., Basingstoke, Hampshire, UK) for *Enterobacteriaceae*,
- Columbia blood agar with 5% sheep blood for *streptococci* (Oxoid Ltd., Basingstoke, UK),
- BBL Enterococcosel agar (BD, Franklin Lakes, USA) for *enterococci*,
- Rogosa Agar (Merck, Darmstadt, Germany) for *lactobacilli*,
- Sabouraud Agar (Merck, Darmstadt, Germany) for *Candida spp*.,
- Schaedler Agar with 5% sheep blood (BD, Franklin Lakes, USA) for anaerobes (*Bacteroides, Clostridium, Fusobacterium*),
- TOS Propionate Agar Base (Liofilchem, Roseto degli Abruzzi, Italy) for *bifidobacteria*.

Incubation durations were set at 37°C for 24 h for aerobic bacteria and 48–72 h for anaerobic bacteria. Colony-forming units (CFU) were computed per 1 g or 1 ml, contingent on the sample type, and converted to percentages for comparative analysis with sequencing data.

Bacterial species identification was authenticated through biochemical tests (API, bioMerieux, l'Etoile, France) and mass spectrometry (MALDI Biotyper, Bruker Scientific LLC, Billerica, MA, USA), adhering to the manufacturer's guidelines. Applying the updated MALDI Biotyper for *In Vitro* Diagnostic (MBT IVD) Library of mass spectra further ensured precision in bacterial identification.

### 2.2 Next-generation sequencing method

All samples analyzed by classical microbial culture were parallelly evaluated by the NGS method. Sequencing was performed in the Center of Postgraduate Medical Education laboratory in Warsaw (CPME). Bacterial DNA was isolated from crushed clinical samples suspended in Schadler liquid using the commercial QIAamp DNA Stool Mini Kit and QIAamp DNA Mini Kit (Qiagen; Hilden, Germany), according to the manufacturer's protocol. Metagenomic analysis of the intestinal and pancreatic microflora was performed using hypervariable fragments of the 16S rRNA gene. Sequencing was performed using Ion 16SMetagenomics Kit (Thermo Fisher Scientific, Waltham, MA, USA) on the Ion Torrent Personal Genome Machine (PGM) platform. Briefly, DNA was subjected to the amplification of 16S rRNA libraries. The concentration of each 16S library was determined by bioanalyzer Agilent 2100 and High Sensitivity DNA Analysis Kit (Agilent^®^ Bioanalyzer^®^, Santa Clara, CA, USA).

The barcoded libraries were prepared using the Ion PGM Template OT2 400 Kit (Fisher Scientific, Waltham, MA, USA) and the Ion OneTouch 2 System (Fisher Scientific, Waltham, MA, USA). A maximum of 32 barcoded 16S samples were sequenced on an Ion 318 v2 chip (Fisher Scientific, Waltham, MA, USA) using the Ion PGM Sequencing 400 Kit (Fisher Scientific, Waltham, MA, USA) according to the manufacturer's instructions.

Unmapped bam files from the PGM were converted into fastq with SamToFastq script (Picard Tools), and the sequences were filtered with a fastq_quality_filter from FASTX-Toolkit (Picard2018toolkit available at http://broadinstitute.github.io/picard/). Bacterial taxa were identified with Mothur version 1.38 (doi: 10.1128/AEM.01541-09). Sequences with more than 10 bases in a homopylymer and with a quality smaller than 20 on a sliding window of 50 bases were removed. Chimeric sequences were removed with chimera vsearch algorithm, implemented in mothur. The 16S rRNA sequences were classified by the Wang method, using the Silva bacterial 16S rRNA database version 138 as a template (doi: 10.1093/nar/gks1219).

Furthermore, in analyzing samples utilizing the NGS method, the CPME laboratory consistently employs standardized S1 and S2 stool specimens obtained from asymptomatic individuals as controls. Notably, the donors (with a median age of 35) refrained from using antibiotics and oral probiotics before stool collection and maintained a normal Body Mass Index (BMI).

### 2.3 Pulsed-field gel electrophoresis

Employing the Pulsed-field Gel Electrophoresis (PFGE) technique, the genomic DNA profiles of select bacterial species were meticulously juxtaposed. This analysis facilitated the discernment of the dynamic shifts within the bacterial flora at distinct anatomical levels of the gastrointestinal tract in the patient diagnosed with MSAP. The comparative DNA profiling focused on bacterial species (*E. coli and E. faecalis*), recurrently isolated from diverse ecological niches.

Bacterial cell suspension and plug preparation adhered to established methodologies outlined in prior publications for *E. coli* (Ejrnaes et al., 2006) and *E. faecalis* (Saeedi et al., 2002). Subsequently, *E. coli* strains' plugs underwent digestion with 25 U of XbaI restriction enzyme (Thermo Fisher Scientific, USA). At the same time, those of *E. faecalis* were subjected to 25 U SmaI, following the manufacturer's guidelines. All isolates underwent analysis utilizing the CHEF-DR III apparatus (Bio-Rad, Hercules, USA). The electrophoretic conditions for *E. coli* were defined as follows: initial switch time 2.0 s, final switch time 35.0 s, run time 21 h, gradient 6 V/cm, temperature 14°C. For *E. faecalis* strains, electrophoresis adhered to the subsequent conditions: initial switch time 5.0 s, final switch time 35.0 s, run time 20 h, gradient 6 V/cm, temperature 14°C. The resulting banding patterns were analyzed rigorously through the GelCompar software (Applied Math, Kortrijk, Belgium).

### 2.4 Analysis of selected bacterial virulence factors by PCR

Quantitative microbiological culture allowed the determination of bacteria types that numerically dominated the remaining bacterial populations. Potentially pathogenic strains from the *Enterobacteriaceae, Enterococaceae*, and *Fusobacteriaceae* families cultured from rectal swabs and stoma content/stool were subjected to further tests. Selected virulence factors responsible mainly for lytic and adherent properties were analyzed for these bacterial species.

In the case of *E. coli*, the PCR reaction was used to check the presence of the following genes: *yggG* and *stcE* (encoding zinc-dependent metalloproteases), *hlyF* (encoding hemolysin), *iucC* (aerobactin siderophore biosynthesis protein), *iutA* (aerobactin receptor), *ibeA* (adhesion to eukaryotic cells), *neu* (colibactin), *pks*+ (polyketide-peptide genotoxin known as colibactin) and sfa/foc (determinants for S fimbrial adhesins). The investigation into the presence of virulence genes within the *Enterococcus* population (*asa1, gelE, hyl, cylA*, and *esp*), responsible for the production of aggregation substance, gelatinase, hyaluronidase, cytolysin, and enterococcal surface protein, was systematically undertaken.

In the case of the *Fusobacterium population*, two genes (*fadA* and *fap2*) were analyzed. *FadA* protein mediates adhesion to and invasion into epithelial and endothelial cells. *Fap2* is responsible for coding outer membrane proteins that induce cell death.

According to the manufacturer's instructions, bacterial DNA isolations were performed using the Genomic Mini kit (A&A Biotechnology, Gdynia, Poland). The PCR reactions were done in a BioRad (California, USA) thermocycler according to previously described methodologies (Kuan et al., 2011; Brandal et al., 2012; Yamamoto et al., 1995; Kaczmarek et al., 2012; Liu et al., 2021; Johnson and Stell, 2000; Vankerckhoven et al., 2004; Kashani et al., 2020; Kaplan et al., 2010). A list of all primers used in the study is presented in Table 1.

**Table 1:** Primers used in the PCR reactions.

**Gene**	**Forward primer (5^′^ → 3^′^)**	**Reverse primer (5^′^ → 3^′^)**	**References**
*yggG*	GAATTCCATATGGACTCCAACGGTCTGCTCAGC	CGCGGATCCTTATTTAATGCCGTCGGCCTTCATGC	Kuan et al., 2011
*stcE*	AGCCCGCGATGATAATAATAAAAT	CGGAGCGGAACCACTGAC	Brandal et al., 2012
*hlyA*	AACAAGGATAAGCACTGTTCTGGCT	ACCATATAAGCGGTCATTCCCGTCA	Yamamoto et al., 1995
*hlyF*	TCGTTTAGGGTGCTTACCTTCAAC	TTTGGCGGTTTAGGCATTCC	Kaczmarek et al., 2012
*iutA*	ATGAGCATATCTCCGGACG	CAGGTCGAAGAACATCTGG	Kaczmarek et al., 2012
*ibeA*	TGAACGTTTCGGTTGTTTTG	TGTTCAAATCCTGGCTGGAA	Kaczmarek et al., 2012
*neuC*	AGGTGAAAAGCCTGGTAGTGTG	GGTGGTACATCCCGGGATGTC	Kaczmarek et al., 2012
*sfa/foc*	CTCCGGAGAACTGGGTGCATCTTAC	CGGAGGAGTAATTACAAACCTGGCA	Kaczmarek et al., 2012
*pks+*	TCACTGTCGTCCCTTTGACG	TAATCGGATCGCCTGACAGC	Liu et al., 2021
*iucC*	AAACCTGGCTTACGCAACTGT	ACCCGTCTGCAAATCATGGAT	Johnson and Stell, 2000
*asa1*	GCACGCTATTACGAACTATGA	TAAGAAAGAACATCACCACGA	Vankerckhoven et al., 2004
*gelE*	TATGACAATGCTTTTTGGGAT	AGATGCACCCGAAATAATATA	Vankerckhoven et al., 2004
*hyl*	ACAGAAGAGCTGCAGGAAATG	GACTGACGTCCAAGTTTCCAA	Vankerckhoven et al., 2004
*cylA*	ACTCGGGGATTGATAGGC	GCTGCTAAAGCTGCGCTT	Vankerckhoven et al., 2004
*esp*	AGATTTCATCTTTGATTCTTGG	AATTGATTCTTTAGCATCTGG	Vankerckhoven et al., 2004
*fadA*	TTCTGCTTCAGCATTCGC	AGTCTTTGAGCTCTTTGAGAT	Kashani et al., 2020
*fap2*	GGGGAAATAGGTCGTTCTGC	CCAACCCCAACACTTTCATC	Kaplan et al., 2010

### 2.5 Evaluation of bacterial tolerance across varying concentrations of bile

In the subsequent phase of our *in vitro* investigations, we systematically examined the impact of varied concentrations of freshly prepared bile (Dehydrate Fresh Bile—Difco Oxgall, Becton-Dickinson, Poland) on the proliferation dynamics of bacterial strains isolated from diverse sample origins within a single patient diagnosed with MSAP.

To execute this, in the formulation of species-specific solid growth media, dehydrated fresh bile was incorporated, resulting in distinct concentrations within the solidified agar: 5, 10, 20, 50, 100, and 200 g/L. The control group consisted of identical culture media lacking the addition of bile. The cultures were cultivated on conventional Petri dishes, 9 cm diameter each, containing 25 ml of agar, and subjected to growth conditions tailored to the unique requirements of individual strains.

The assessment of bacterial growth rates was conducted through a semi-quantitative scale, wherein 3 denoted heavy growth, 2 indicated moderate growth, 1 represented poor growth, and 0 signified an absence of any discernible growth.

### 2.6 Impact of bile on the generation of gaseous metabolites by specific bacterial strains

**Figure 1:**
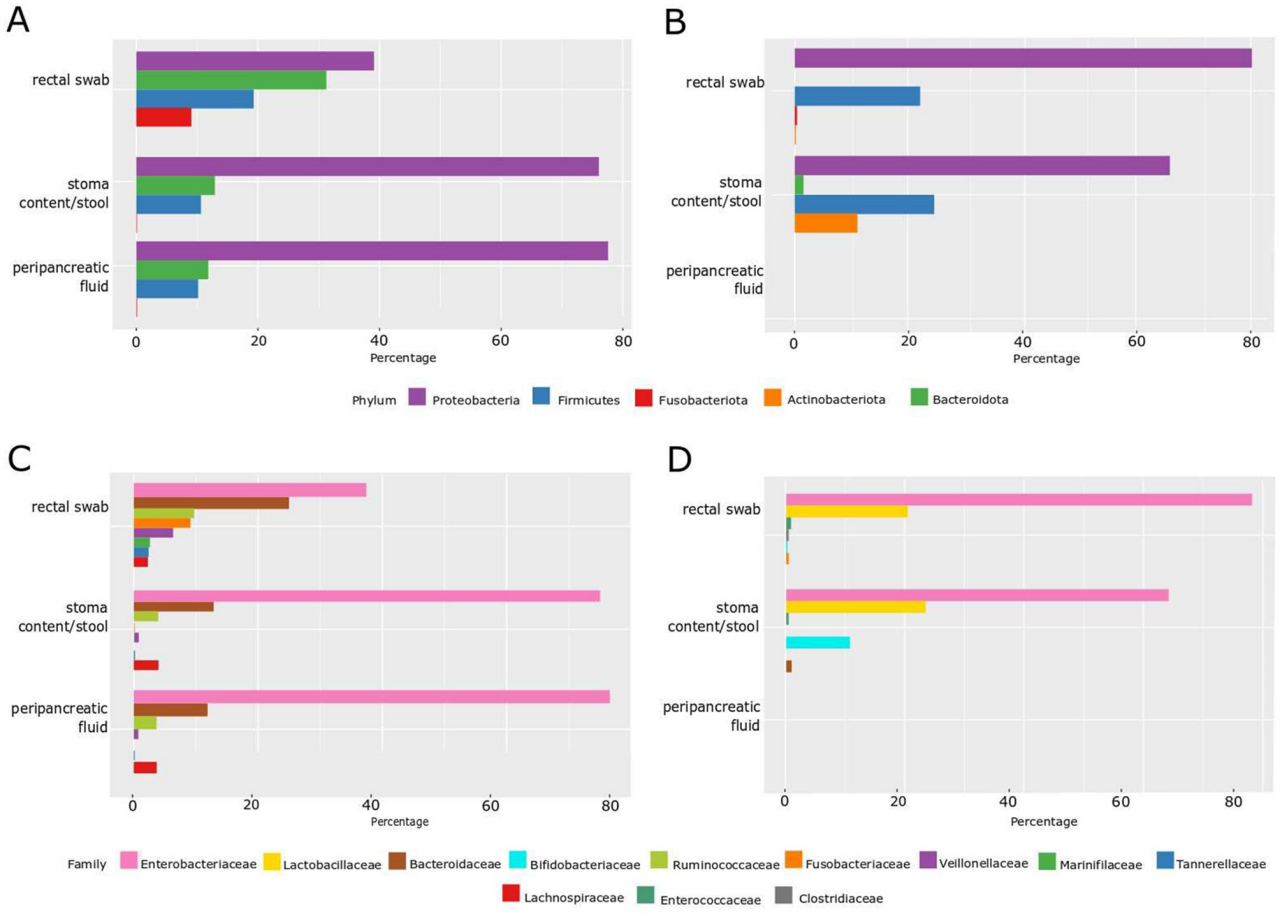
Percentage of microbiota distribution by phylum and family level by the NGS method and by the classical culture method. **(A)** Phylum level—NGS; **(B)** phylum level—classical culture method; **(C)** family level—NGS; **(D)** family level—classical culture method.

Selected bacterial strains (potentially pathogenic strains from the *Enterobacteriaceae, Enterococaceae*, and *Fusobacteriaceae* families) were assessed for their ability to release gaseous post-culture metabolites depending on the presence or absence of bile addition in the growth medium. Gaseous bacterial metabolites were collected in suitable 0.6 l gas sampling bags (Tedlar gas sampling bag; Jensen Inert Products, NW, Coral Springs, Florida) for gas chromatography measurements. The bags were tightly connected (using appropriate valves—Figure 1) with a 250 ml culture flask filled with 230 ml of the appropriate solid medium (selection of the medium and culture conditions adapted to the strain—described in the materials and methods). A selected concentration of bile, i.e., 20 g/L, was added to each of these media. This concentration was chosen after analyzing the bile tolerance results of all tested bacteria. Flasks with the same agar media without adding bile served as controls.

Subsequently, 10 ml of a liquid 24-h culture of selected bacteria (population number was about 1 × 10^8^ CFU/ml) was poured onto the surface of the solidified agar in individual flasks. The culture inoculum flasks were securely connected to a specialized gas-collection bag through valves. The cultivation process spanned 5 days, following which qualitative and quantitative analyses of gas samples were conducted utilizing a GC-MS chromatograph QP2020 (Shimadzu, Kyoto, Japan) equipped with a thermal conductivity detector (TCD). An MS line was equipped with an HP-PLOT/U column (Agilent, J&W, 0.530 mm diameter, 30 m length). A TCD line used to analyze hydrogen concentration was equipped with a 5 Å molecular sieve-packed column. The MS line was supplied with a helium carrier gas with a flow rate of 377.5 ml·min^−1^, while the TCD line was supplied with a nitrogen carrier gas with a 15 ml·min^−1^ flow rate. The oven temperature during measurements was set at 35°C. Before each measurement, the molecular sieve and the chromatographic column were conditioned at 150°C for 600 s. Quantitative analyses were performed on calibration curves based on certified gas mixtures supplied by Air Liquide Poland. Calibration curves and quantitative analyses were performed on areas of peaks related to particular gases.

## 3 Results

Based on the classical culture method, we isolated 28 viable bacterial strains from one MSAP patient (11 from rectal swabs and 17 from stoma content/stool). However, we did not detect any viable bacteria in the peripancreatic fluid (the cumulative result is shown in Table 2).

**Table 2:** Bacteria isolated from a patient with MSAP by using qualitative and quantitative classical culture method.

**Material**	**Phylum**	**CFU/g or CFU/ml**	**Family**	**Species**	**CFU/g or CFU/ml**
Rectal swab	*Proteobacteria*	4.9 × 10^8^	*Enterobacteriaceae*	• *Escherichia coli* • strains: 14, 25	4.9 × 10^8^
	*Firmicutes*	1.37 × 10^8^	*Enterococcaceae*	• *Enterococus faecalis* • strain 34	5 × 10^6^
			*Lactobacillaceae*	• *Lactobacillus salivarius* • strains:16, 36 • *Lactobacillus plantarum* • strain: 17	1.3 × 10^8^
			*Clostridiaceae*	• *Clostridium perfringens* • strain: 37	2 × 10^6^
	*Fusobacteriota*	4 × 10^6^	*Fusobacteriaceae*	• *Fusobacterium nucleatum* • strain: 30	4 × 10^6^
	*Actinobacteriota*	1 × 10^6^	*Bifidobacteriaceae*	• *Bifidobacterium adolescentis* • strain: 38 • *Bifidobacterium dentium* • strains: 39, 40	1 × 10^6^
Stoma content/stool	*Proteobacteria*	6 × 10^8^	*Enterobacteriaceae*	• *Escherichia coli* • strains: 10, 18, 33	3 × 10^8^
				• *Morganella morganii* • strain: 9	3 × 10^6^
	*Firmicutes*	2.24 × 10^8^	*Enterococcaceae*	• *Enterococcus faecalis* • strain: 19, 26	4 × 10^6^
			*Lactobacillaceae*	• *Lactobacillus salivarius* • strains: 11, 21 • *Lactobacillus kalixensis* • strain: 12 • *Lactobacillus plantarum* • strains: 22, 28	2.2 × 10^8^
	*Actinobacteriota*	1 × 10^8^	*Bifidobacteriaceae*	• *Bifidobacterium dentium* • strains: 13, 24, 31, 32 • *Bifidobacterium adolescentis* • strain: 23	1 × 10^9^
	*Bacteroidota*	1 × 10^7^	*Bacteroidaceae*	• *Bacteroides fragilis* • strain: 29	1 × 10^7^
Peripancreatic fluid		No growth

However, NGS confirmed the presence of bacterial DNA in all samples, including peripancreatic fluid, which would also indicate the presence of bacteria or bacterial DNA in this place (Figures 1A, C).

Additionally, the NGS technique verified a greater abundance of phylum and bacteria families in rectal swabs compared to stoma content/stool and peripancreatic fluid, with these two last samples showing more remarkable similarity to each other both in phylum (*Proteobacteria*—over 75%, *Bacteroidota*—over 12%, *Firmicutes*—about 10%) and in family percentage distribution (*Enterobacteriaceae*—about 75%, *Bacteroidaceae*—about 12% and *Ruminococcaceae* and *Lachnospiraceae*—about 5%). Furthermore, NGS verified the presence of the *Fusobacteriota* phylum and the *Fusobacteriaceae* family, constituting ~10% of the rectal swab composition. The other two samples did not identify these microbial components—stoma content/stool and peripancreatic fluid. The concordance of these findings was corroborated through culture methods, where viable *Fusobacterium nucleatum* was exclusively identified in the rectal swab.

**Figure 2:**
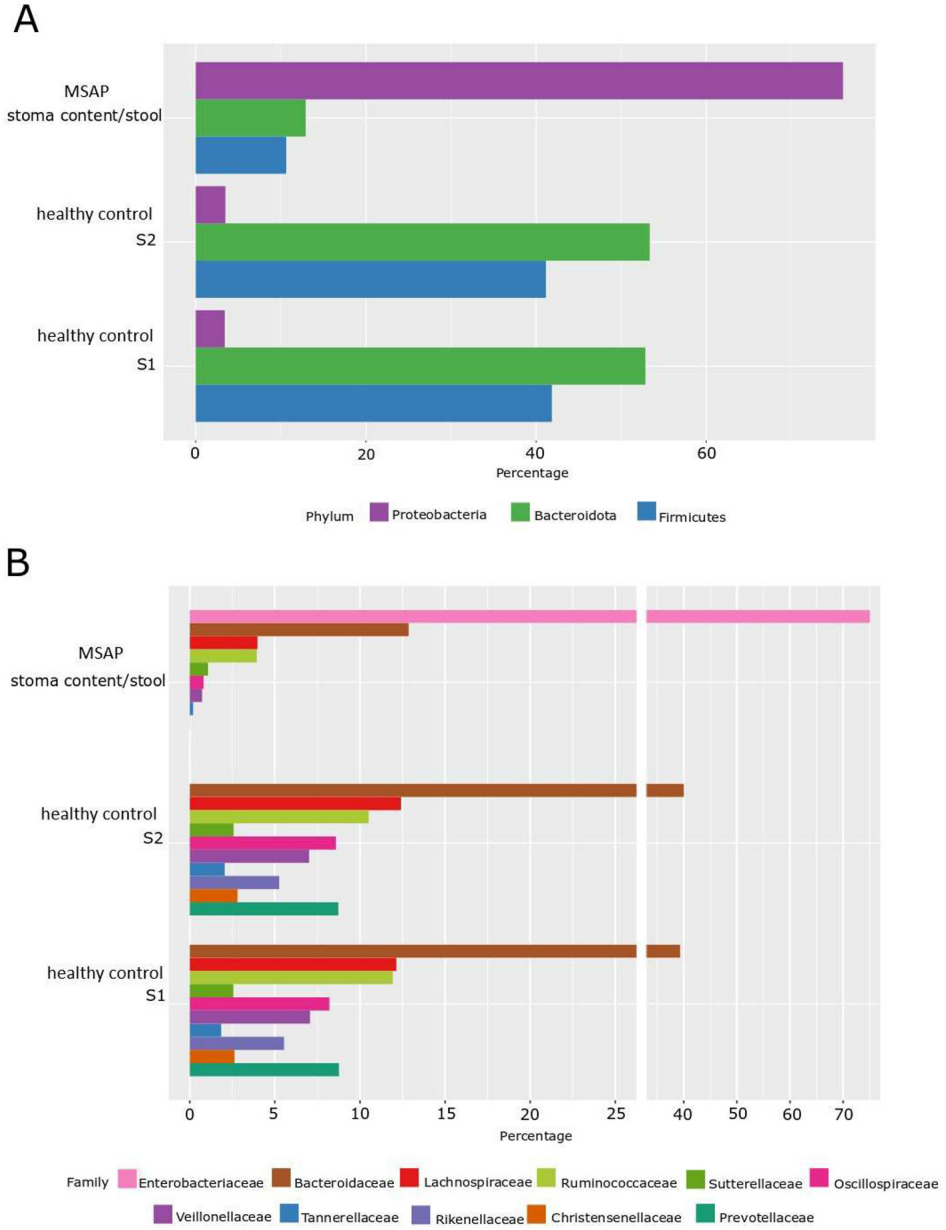
Percentage distribution of microbiota by phylum and family level determined by the NGS method. **(A)** Phylum level—NGS. **(B)** Family level—NGS; S1—healthy control patient 1, S2—healthy control patient 2, MSAP- moderately severe acute pancreatitis.

Utilizing the NGS method, a comparative analysis of the microflora distribution percentage at both the phylum and family levels was facilitated in stool samples obtained from two healthy donors, S1 and S2 (internal laboratory controls). This comparative assessment was conducted in relation to the stoma content/stool derived from a patient diagnosed with MSAP. We observed fascinating differences; firstly, in healthy control donors, the distribution of phylum is dominated mainly by anaerobic and microaerophilic bacteria, i.e., *Bacteroidota* (over 42%), then *Firmicutes* (over 30%), *Proteobacteria* (about 5%; Figure 2A).

In the case of the MSAP patient, aerobic flora had a prevailing presence, notably marked by the dominance of the *Proteobacteria phylum*, comprising ~77% of the microbial composition. Subsequently, the *Bacteroidota* phylum accounted for ~12%, followed by the *Firmicutes* phylum at ~10%. Furthermore, within the *Proteobacteria* group, the *Enterobacteriaceae* family, representing aerobic Gram-negative bacilli, emerged as the predominant family.

This observed shift suggests a potential alteration in oxidative-reductive (ox-redox) conditions within the digestive tract. Specifically, the ongoing inflammatory process may have induced a transition from anaerobic to aerobic conditions. Noteworthy is the significant change in the ratio between the two phyla, with anaerobic *Bacteroidota* surpassing aerobic *Proteobacteria* at 8:1 in the control group. In contrast, in the MSAP patient, the ratio shifted markedly to almost 1:7, indicating a noteworthy shift in microbial composition (Figures 2A, B).

### 3.1 PFGE

PFGE was performed for all *E. coli* (*n* = 5) and *E. faecalis* (*n* = 3) strains isolated from both rectal swabs and stoma content/stool. Analysis of *E. coli* isolates revealed three different genetic profiles (pulsotypes), which confirmed three separate *E. coli* strains (10, 25, and 33).

**Figure 3:**
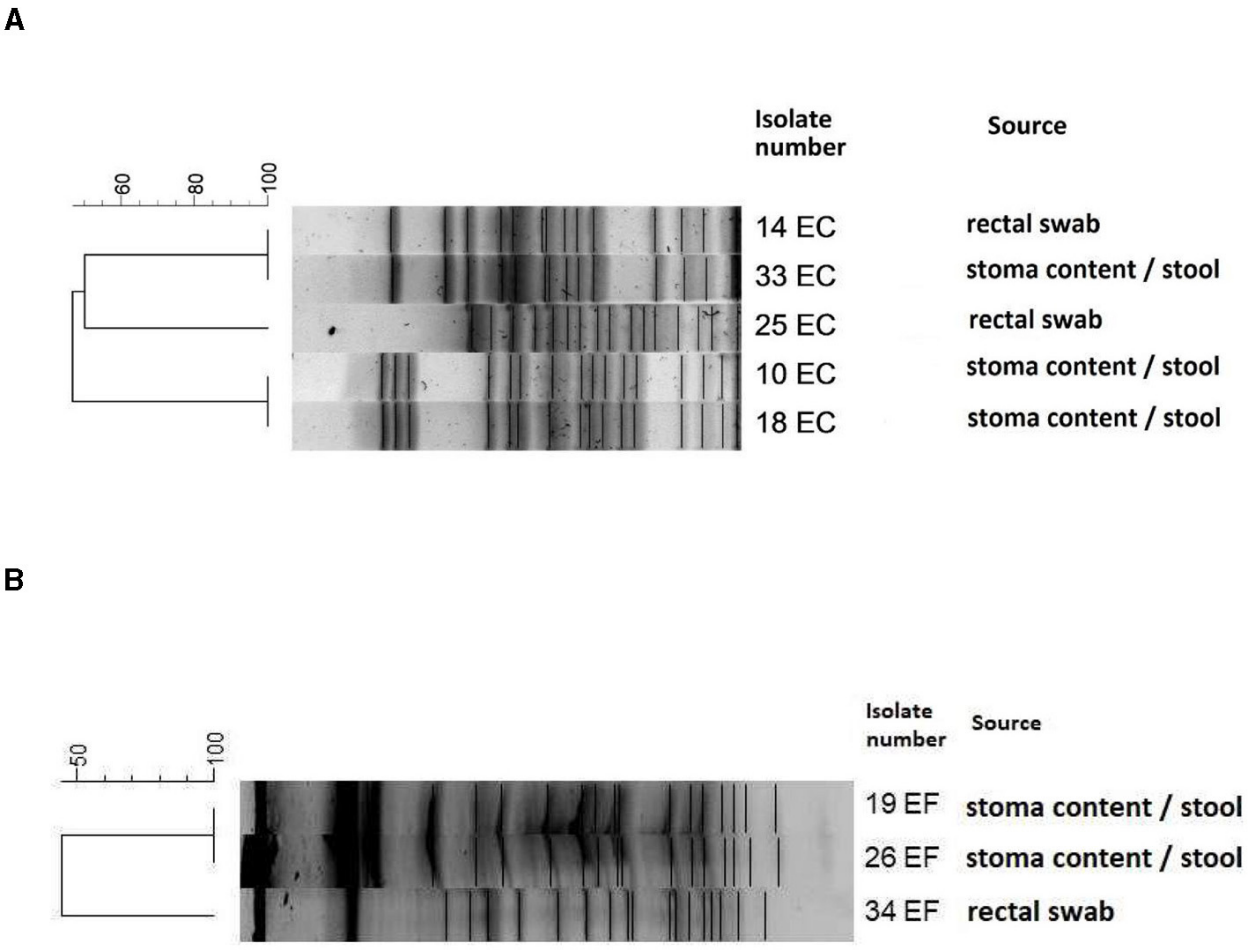
Dendrogram of PFGE patterns showing the genetic relatedness of *E. coli* strains **(A)** and *E. faecalis* strains **(B)**. The scale shows the percentage of similarity among different strains. Dice coefficient and unweighted pair group method with arithmetic mean UPGMA was used for cluster analysis; isolates with more than 95% similarity were clustered together as identical. *Escherichia coli* 14 and 33, 10 and 18 were identical pulsotypes. Among the *Enterococcus faecalis* strains, 19 and 26 were identical pulsotypes.

*E. coli* 18 had an identical profile as *E. coli* 10, and *E. coli* 14 had an identical profile as *E coli* 33. Among three analyzed *E. faecalis* isolates, two strains isolated from stoma content/stool (*E. faecalis* 19 and *E. faecalis* 26) had the same pulsotypes, while *E. faecalis* 34 isolated from rectal swabs had a unique genetic profile. Detailed results are presented in Figure 3.

### 3.2 PCR—virulence genes

In all *E. coli* strains, we detected the presence of genes for hemolysin (*hlyF*), aerobactin (*iutA*), aerobactin siderophore biosynthesis protein (*iucC*), and polyketide-peptide genotoxin known as colibactin (*pks*+).

Strains of *E. faecalis* possessed genes encoding hydrolysis of gelatin, collagen, casein, hemoglobin (*gels*), and a gene responsible for increasing biofilm production (*esp*).

We detected *fadA* gene, responsible for adhesion to and invasion into epithelial and endothelial cells in the *F. nucleatum* strain isolated from a rectal swab. The detailed virulence profile of all the tested strains is shown in Table 3.

**Table 3:** Distribution of different virulence genes in tested strains.

**Material**	***S*train no**.	**Virulence genes**									
***E. coli***		***ygg*** **G**	***stc*** **E**	***hly*** **A**	***hly*** **F**	***iuc*** **C**	***iut*** **A**	***ibe*** **A**	***neu***	***pks***+	***sfa*** **/** ***foc***
Rectal swab	*E.coli* 14	0	0	0	1	1	1	0	0	1	0
	*E.coli* 25	0	0	0	1	1	1	0	0	1	0
Stoma content/stool	*E. coli* 10	0	0	0	1	1	1	0	0	1	0
	*E. coli* 18	0	0	0	1	1	1	0	0	1	0
	*E. coli* 33	0	0	0	1	1	1	0	0	1	0
***E. faecalis***		***asa***	***gel***	***hyl***	***cyt***	***esp***					
Rectal swab	*E.faecalis* 34	0	1	0	0	1					
Stoma content/stool	*E.faecalis* 19	0	1	0	0	1					
	*E.faecalis* 26	0	1	0	0	1					
***F. nucleatum***		***fad*** **A**	***fap*** **2**								
Rectal swab	*F.nucleatum* 30	1	0								

1 (presence); 0 (absence); hlyA, encoding hemolysin; hlyF, induced autophagy in eukaryotic cells; stcE, encoding metalloprotease; iucC, aerobactin siderophore biosynthesis protein; iutA, aerobactin receptor; ibeA, responsible for adhesion to eukaryotic cells; neu, pks, polyketide-peptide genotoxin known as colibactin, pks islands have been significantly associated with bacteremia; sfa/foc, determinants for S fimbrial adhesins; asa1, encoding surface aggregating protein; gelE, gelatinase; cylA, cytolysin; hyl, hyaluronidase; esp, extracellular surface protein; fadA, adhesion to and invasion into epithelial and endothelial cells; fap2, familial adenomatous polyposis 2, outer membrane protein, induced cell death.

### 3.3 Bacterial fresh bile tolerance

Our *in vitro* investigations examined the impact of varying concentrations of fresh bile on the growth rates of strains isolated from diverse sample origins within a single patient diagnosed with MSAP. Notably, strains belonging to the *Enterococcus* spp. exhibited the highest tolerance to bile, showcasing resilience across a broad spectrum of concentrations ranging from 5 to 200 g/L. Conversely, *F. nucleatum* displayed comparatively lower resistance, manifesting limited growth, particularly at a bile concentration of 50 g/L.

Interestingly, all tested strains demonstrated robust growth at a bile concentration of 20 g/L, signifying a pivotal threshold. Consequently, this concentration was strategically chosen for subsequent studies, owing to its consistent and favorable impact on the growth behavior of the microbial strains under examination.

### 3.4 The influence of fresh bile concentration (20 g/L) on gaseous metabolite production

**Figure 4:**
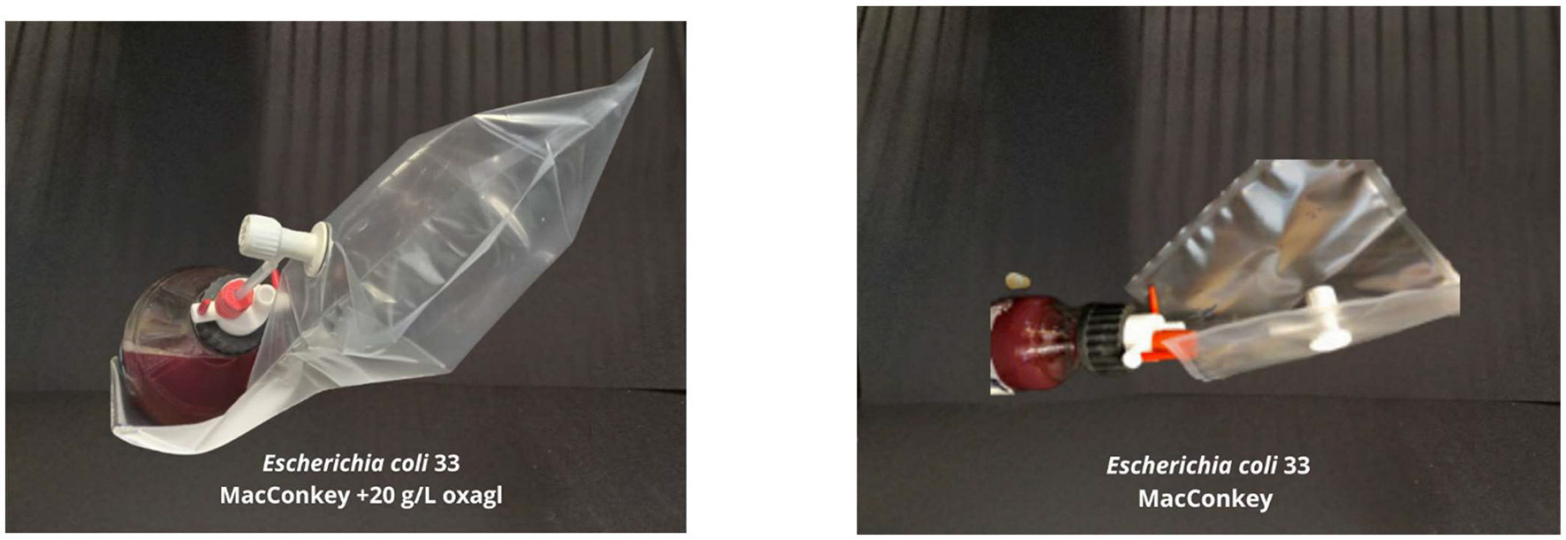
The effect of bile on gaseous metabolite production. **(Left)** *E.coli* 33 was grown on MacConkey agar medium with 20 g/L bile—a visible gas-filled bag. **(Right)** The same strain of *E. coli 33* is grown on MacConkey agar with no bile—much lower gas production was observed.

The capacity for generating gaseous metabolites was assessed across all extant bacterial strains delineated in Table 4. Among these, the *E. coli 33* sourced from stoma content/stool and *E. coli 14* obtained from rectal swabs demonstrated proliferation within the medium containing 20 g/L bile. These strains exhibited a sufficient production of gaseous metabolites, evidenced by visible inflation of the collection bag, as depicted in Figure 4. However, thrice-repeated gas measurements showed that *E. coli 33* released a markedly higher quantity of gas metabolites than *E. coli 14*. Consequently, we proceeded exclusively with the *E. coli 33* for subsequent chromatographic examinations. Conversely, no discernible gaseous emissions were noted within the gas sampling bags from the remaining bacterial strains enumerated in Table 4.

**Table 4:** Measurement of bacterial tolerance to the fresh bile.

**Strain no**	**Material**	**Tolerance to bile**					
		**5 g/L**	**10 g/L**	**20 g/L**	**50 g/L**	**100 g/L**	**200 g/L**
*E. coli* 10	Stoma content/stool	3	3	3	3	3	0
*E. coli* 18	Stoma content/stool	3	3	3	3	3	0
*E. coli* 33	Stoma content/stool	3	3	3	3	3	0
*E. coli* 14	Rectal swab	3	3	3	3	3	0
*E.coli* 25	Rectal swab	3	3	3	3	3	0
*E. faecalis* 19	Stoma content/stool	3	3	3	3	3	1
*E. faecalis* 26	Stoma content/stool	3	3	3	3	3	1
*E. faecalis* 34	Rectal swab	3	3	3	3	3	1
*F. nucleatum* 30	Rectal swab	3	3	3	1	1	0

Measurement of bacterial growth was assessed semi-quantitatively using the following scale: 3-intense growth, 2-medium growth, 1-poor growth, 0-no growth at all.

### 3.5 The qualitative and quantitative gas sample analyses performed with GC-MS chromatograph for *E. coli* 33

**Figure 5:**
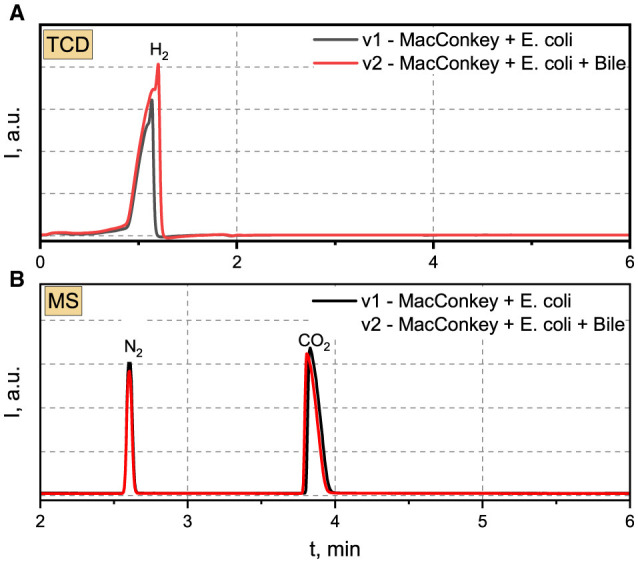
**(A)** TCD and **(B)** MS chromatograms recorded for analyzed gas samples produced by E.coli 33.

The gas chromatogram, acquired utilizing the TCD and featuring a retention time (t_R_) of 1.14 min, reveals conspicuous peaks indicative of hydrogen presence in the scrutinized samples (refer to Figure 5). Evident chromatographic patterns signify a heightened concentration of hydrogen within the analyzed samples, ranging from 13.78% (v1) to 23.02% (v2), as delineated in Table 5. The cumulative volume of the collected gas phase was slightly higher in the system incorporating bile. The average hydrogen production rate by *E. coli* 33 within MacConkey agar manifested as 3.12 and 5.43 ml/day in electrolytes absent and present with bile, respectively. We deduce that the inclusion of bile in the system increases hydrogen production, stemming from the formation of the hydrogen gas phase due to the combination of atomic hydrogen within MacConkey agar.

**Table 5:** The total volume of gas samples produced after 6 days by *E. coli 33* placed in MacConkey agar and MacConkey agar containing bile.

**Sample**	**Description**	**V_c_, ml**	**H_2_, %**	**CO_2_, %**
v1	MacConkey + *E. coli* 33	135.8	13.78	27.71
v2	MacConkey + *E. coli* 33+ Bile	141.5	23.02	26.77

The Mass Spectrometry (MS) chromatograms revealed the presence of nitrogen and carbon dioxide in the examined samples. Nitrogen in the gas sample derived from a “dead space” within the MacConkey agar during the initial stages of the experiment. Conversely, CO_2_ was generated by *E. coli* 33. The intensity of nitrogen peaks suggests a closely comparable concentration irrespective of the applied environmental conditions. A parallel scenario was noted for CO_2_, with concentrations reaching 27.71 and 26.77% for v1 and v2, respectively. The generation of CO_2_ in MacConkey agar, devoid and in the presence of bile, amounted to 6.28 and 6.31 ml/day, respectively.

## 4 Discussion

The gut microbiota composition is inherently individualized, exhibiting a distinctive profile for each person. On a normative basis, it encompasses an excess of 1,500 distinct species and spans over 50 phyla. The superior abundance is typically attributed to Bacteroidetes, succeeded by Firmicutes, along with other prevalent phyla, including Proteobacteria, Actinomyces, Actinobacteria, Fusobacteria, and Verrucomicrobia (Qin et al., 2010).

In recent years, there has been a surge in comprehensive investigations on intestinal microbiota composition among individuals afflicted with diverse diseases, encompassing acute, chronic, and autoimmune conditions (Nagata et al., 2022; De Luca and Shoenfeld, 2019; Li et al., 2020).

Shifts in the gut microbiota may also be involved in the pathogenesis of pancreatic disease (including AP), chronic pancreatitis, or pancreatic cancer (PC). Developing screening methods for early pancreatic disease diagnosis is a priority. Metabolic modifications that occur in the functioning of the pancreas affect the composition of the intestinal microbiota. Hence, potential non-invasive biomarkers can be sought among the fecal bacteria and their metabolic products. Such investigations are being carried out toward PC diagnosis (Nagata et al., 2022; Kartal et al., 2022), early hepatocellular carcinoma diagnosis (Li et al., 2020; Ren et al., 2019), and differentiating patients with pancreatic ductal adenocarcinoma and autoimmune pancreatitis (Zhou et al., 2021).

The material commonly employed for investigating the gastrointestinal microbiota (relying on analyzing the 16S rRNA bacterial gene sequence and culturing techniques) predominantly comprises stool samples. However, our study placed particular significance on peripancreatic fluid as the primary material. Herein, we successfully demonstrated the presence of bacterial DNA utilizing NGS despite the absence of viable bacterial growth on the culture medium. This underscores the unique utility of peripancreatic fluid in our research, allowing for a comprehensive exploration of microbial genetic material even in instances where traditional culture methods prove inconclusive.

Obtaining stool samples from patients with gastrointestinal diseases, including AP, can be challenging because patients frequently follow a restricted dietary regime and suffer from constipation. Microbiological cultures can yield falsely negative results when patients undergo broad-spectrum antibiotic therapy. Other materials, such as biopsies, are limited; they are available in selected diseases, e.g., necrotizing pancreatitis. Challenges associated with procuring materials for microbiological assessment may occasionally be misleading and questionable.

The current study by Bassis et al. (2017) indicates the possibility of using the NGS technique for microbiological analysis of rectal swabs as an appropriate alternative for patients with acute pancreatitis, for whom obtaining other clinical samples, including stools, is a significant problem.

The structure of the bacterial communities in the Bassis study was similar between freshly passed stool and rectal swabs collected at different time points in each patient. Rectal swabs are a relatively uncomplicated sample to collect, do not require patient preparation, and can be easily transported to the laboratory. Therefore, rectal swabs may be an acceptable and practical substitute for stool collection and fecal microbiota analysis, which our study confirms.

In our investigation, the patient had previously experienced diverticulitis 6 months prior, subsequently undergoing Hartmann's surgery involving colostomy. By applying NGS analysis on bacterial microflora, we successfully showcased a correlation between the microbial structures found in samples from the stoma and those in the peripancreatic fluid, which may indicate the migration of bacteria from the intestinal lumen, where the diverticulitis process was taking place, to the pancreas and peripancreatic space (resulting in acute pancreatitis). However, the progression was relatively slow, as several months passed from the establishment of the stoma to the manifestation of AP symptoms.

In the rectal swab specimen obtained from a patient diagnosed with MSAP, a discernibly elevated microbial population was observed compared to samples derived from individuals in good health. Notably, there was a pronounced prevalence of aerobic bacteria belonging to the *Proteobacteria* genus, surpassing the presence of anaerobic and microaerophilic bacterial counterparts. This discovery holds significant scientific merit, particularly in light of Zhou et al.'s (2020) research, which concurrently demonstrated the selective enrichment of *Proteobacteria* in the intestinal microbiota of individuals affected with pancreatic cancer as opposed to their healthy counterparts.

The patient diagnosed with MSAP exhibited elevated relative abundances of potentially pathogenic bacteria (Enterobacteriaceae and Enterococcus), as revealed by both NGS and culture studies. In their recent investigation, Sexton et al. highlighted that any perturbations in the human microbiome have the potential to instigate immune processes, including inflammation, thereby contributing to the onset of diseases (Sexton et al., 2022).

The prevalence of aerobic bacteria in the intestinal milieu of AP patients may instigate an unfavorable redox potential, thereby impacting intestinal epithelial cells. Imbalances in oxidative stress can manifest as intestinal inflammation and compromise the integrity of the intestinal barrier. Notably, microorganisms, particularly Gram-negative rods (through endotoxin activity,) can incite inflammatory responses, enhance the recruitment of pro-inflammatory cells, stimulate cytokine secretion, elevate susceptibility to oxidative stress, perturb energy dynamics, and inflict damage to DNA, as elucidated by Serra et al. (2018).

The increase in the Proteobacteria population presumably causes a proliferation in the diversity of *Enterobacteriaceae* and *Enterococcaceae*, which is reflected in the isolation of three different *E. coli* and two different *E. faecalis* genotypes in the examined patient. All three isolated *E. coli* strains possessed the same set of virulence factors: *hlyF* (hemolysin-inducing autophagy in eukaryotic cells), *iucC* (aerobactin siderophore biosynthesis protein), *iutA* (gene helping in iron acquisition in *E. coli* by producing siderophores) and *pks*+ (gene coding genotoxin colibactin that induces DNA damage, cell cycle arrest and mutations in eukaryotic cells) (Sarowska et al., 2019). Similarly, both isolated *E. faecalis* strains exhibited the presence of the same virulence genes (*gelE* and *esp*). The gelatinase, encoded by the *gelE* gene, plays a crucial role in facilitating the translocation of *Enterococcus faecalis* across human enterocytes, thereby promoting microbial invasion. Simultaneously, the enterococcal surface protein (*Esp*) is implicated in the adhesion and colonization processes, underscoring its significance in the initial stages of host-microbe interactions (Kiruthiga et al., 2020). In the available literature, no reports show the relationship between specific virulence gene combinations in the mentioned bacterial species and their ability to cause AP. Nevertheless, all identified virulence factors of *E. coli* and *E. faecalis* in our study can be helpful for the bacteria to colonize, multiply, and survive in the peripancreatic space. In 2003, Ammori identified these two species (stemming from the intestinal microbiota) as contributors to necrotic infections in pancreatic tissue (Ammori, 2003). Moreover, Tan et al. (2015) showed a significant increase in the *Enterobacteriaceae* and *Enterococcus* population in patients with AP compared to the control group in their multi-hospital prospective clinical study. Therefore, the intestinal flora may play an essential role in the development of AP. Increased levels of potentially pathogenic bacteria, such as *Enterobacteriaceae* and *Enterococcus*, may contribute to intestinal microbiota dysbiosis and dysfunction of the intestinal barrier, eventually leading to bacterial translocation (Chen et al., 2011; Bauer et al., 2002; Manichanh et al., 2006). Studies have revealed that intestinal mucosal barrier injury is one of the significant complications of AP. A meta-analysis revealed that 59% of AP patients had associated intestinal barrier injury with increased intestinal mucosal permeability, leading to intestinal bacterial translocation, pancreatic tissue necrosis, and infection (Capurso et al., 2012). Also, in our *in vitro* studies conducted on human Caco-2 cell lines, we observed that an increased population of *E. coli* (over 1 × 10^7^ CFU/ml) reduced the secretion of occludin and zonulin-tight junction proteins produced by intestinal epithelial cells, which may directly affect the intestinal barrier tightness (unpublished data).

Interestingly, using the NGS and the classical culture methods, we demonstrated the presence of many *Fusobacteria* in the rectal swab of a patient with MSAP, which we did not find in the stools of healthy donors (samples S1 and S2). *F. nucleatum* is a Gram-negative anaerobic bacterium that lives in the oral cavity and produces lipopolysaccharides and other virulence factors, including *FadA* (adhesin) and *Fap2* (outer membrane protein), which play a significant role in causing local diseases. *F. nucleatum* promotes inflammation (a potent stimulator of inflammatory cytokines) and binds to or attacks many cell types, including oral and colon T cells, keratinocytes, and macrophages (Kinder Haake and Lindemann, 1997). The *FadA* adhesin enables *F. nucleatum* to bind host cells in the pancreatic environment by interacting with cadherins (Sun et al., 2019).

The interaction between *F. nucleatum* and natural killer (NK) cells is mediated by the *Fab2* protein, which binds to TIGIT receptors on the NK cell surface. This binding event leads to the impairment of NK cell function, specifically by suppressing their natural cytotoxicity. Consequently, this bacterial interference renders NK cells unable to effectively target and attack cancer cells, as substantiated in colon cancer studies (Gur et al., 2015).

Contemporary medical literature has prominently explored the correlation between *Fusobacterium* species and acute intra-abdominal infections such as peritonitis, hepatic abscesses, and AP, mainly when linked to previous diverticulitis episodes (Brook and Frazier, 2000; Bawa et al., 2022). In our patient's case, isolating viable *F. nucleatum* rods from the rectal swab suggests a potential direct association of this bacterial species with a prior episode of acute diverticulitis. Bawa et al. (2022) documented a parallel clinical scenario where recurrent diverticulitis with abdominal pain was linked to *F. nucleatum* upon detailed microbial investigation. While confirmation is speculative, it is plausible that diverticulitis initiated by *Fusobacterium* could have disseminated or induced secondary dysbiosis in the peripancreatic space. This speculation gains support from (Del Castillo et al. (2019), who reported a heightened relative abundance of *Fusobacterium spp*. in samples from pancreatic cancer patients. Furthermore, Mitsuhashi et al. demonstrated correlations between the quantity of *F. nucleatum* and reduced survival rates in patients with pancreatic ductal adenocarcinoma (PDAC) (Mitsuhashi et al., 2015).

There is also promising research on using these microorganisms as biomarkers in colorectal cancer screening. Guo et al. (2018) showed that testing the ratio of *F.nucleatum* to *Bifidobacterium* allows the detection of colorectal cancer with 84.6% sensitivity and 92.3% specificity.

Consequently, monitoring the elevation in *Fusobacterium* numbers through comprehensive analyses such as NGS and traditional culture methods in rectal swabs and other relevant samples may be a valuable indicator for tracking inflammation progression. Such assessments could be markers for evaluating the risk of the patient's deteriorating condition and influencing survival outcomes.

Bile duct obstructions and gallstone migration cause gallstone-induced pancreatitis. Bile duct and/or pancreatic duct obstruction may lead to a local increase in bile pressure, resulting in irregular digestive enzyme activation and local inflammation. Consequently, it may damage pancreatic tissue and cause uncontrolled bile leakage into the peripancreatic space (Wang et al., 2009).

Bile is an essential substrate added to many bacterial media, which can directly influence the shift of the microbial balance toward species resistant to this factor. *Streptococci* of the *Enterococcus* genus and Gram-negative rods of the *Escherichia* genus are among the bacteria whose population growth and maintenance of population viability strictly depend on the presence of bile in the medium. Therefore, an increase in the *E. coli* population in the peripancreatic environment under the influence of bile may be a sufficient agent causing intestinal inflammatory process progression as it may lead to the accumulation of lipopolysaccharides (LPS). This endotoxin strongly stimulates immune system cells but may also lead to robust gaseous metabolite production [mainly hydrogen (H_2_)]. Based on our results, the production of gaseous metabolites under the influence of bile is a strain-dependent feature because only one strain of *E. coli 33* isolated from stool (accumulated in the stoma bag) showed active production of gaseous metabolites. In contrast, the other three *E. coli* strains did not have such spectacular activities despite being cultured in the same conditions.

What function can such hydrogen accumulation play in the inflamed peripancreatic space? The role of hydrogen accumulation in the inflamed peripancreatic space is not fully explained in current literature, particularly regarding the biological functions of gaseous metabolites generated by both host cells and bacteria during the initiation, maintenance, and resolution of inflammatory processes in the intestines. Some research groups exploring the biological functions of different gases have noted that hydrogen (H_2_), given its reducing properties, may undergo conversion into hydrogen sulfide (H_2_S). Considering that many biochemical processes can occur in human bodies, such amounts of hydrogen of a reductive nature may significantly affect enzymatic processes through specific changes in disulfide bonds and inactivation of trypsin (He et al., 2023). Mee et al. (1965) reported that atomic hydrogen might attack disulfide bonds, forming sulphydryl groups or hydrogen sulfide and destroying tryptophan (Wimmer and Zarevúcka, 2010). Hydrogen sulfide (H_2_S) exhibits dual roles, as at low physiological concentrations, it [alongside nitric oxide (NO) and carbon monoxide (CO)] can function as a signaling molecule that regulates vasodilation. This activity contributes to a reduction in blood pressure.

On the other hand, other authors indicate that H_2_S also adversely affects intestinal peristalsis, which is highly unfavorable in patients with AP. Moreover, H_2_S has a toxic effect on intestinal epithelial cells and causes clinical symptom exacerbation in patients with ulcerative colitis. H_2_S also exerts a negative impact on carcinogenesis processes. Studies in cell cultures and a mouse model have shown that colon cancer cells produce large amounts of H_2_S, which they then use to produce energy, divide, and grow the tumor. Hydrogen sulfide is synthesized by host cells in various physiological systems, including the circulation, gastrointestinal tract, and nervous system. Its production involves two enzymes, and vitamin B6 is a cofactor in this process. Studies, such as those conducted on rats with experimental sepsis induced by colon ligation and puncture, as well as toxic shock triggered by lipopolysaccharide administration, have confirmed an upsurge in H_2_S production within blood vessels, aligning with a significant decrease in blood pressure (Liu et al., 2017). The hydrogen sulfide generated, whether in its gaseous state or dissociated forms (HS–, S2–), can induce damage to the intestinal mucosa and exert cytotoxic effects on intestinal epithelial cells (Bełtowski, 2004).

Bacteria inhabiting the appropriate ecological niches do not necessarily penetrate deeply into the host's tissues as a living cell to initiate acute inflammation. Only bacterial post-culture metabolites such as LPSs, peptidoglycan, exopolysaccharide, enzymes, metalloproteases, or gases are sufficient for the occurrence of acute or chronic inflammatory processes.

Understanding the primary causes and mechanism of the pathogenesis and progression of AP is essential to facilitate early diagnosis and treatment and avoid a course leading to severe disease onset (Patel et al., 2021).

Our study indicates the possibility of active participation of bile in qualitative and quantitative changes in the composition of the microbiota in the peripancreatic space and the connection between released bile and the bacterial production of gaseous metabolites.

## 5 Limitations

This study investigated the gastrointestinal microbiota of a single patient who developed acute pancreatitis following a prior episode of diverticulitis, a condition described by surgeons as exceptionally rare. Despite being a single clinical case, we were able to collect a substantial number of parallel samples, enabling a comparative analysis of classical culture methodologies and next-generation sequencing (NGS). Notably, we successfully cultured bacteria potentially implicated in the pathomechanism of acute pancreatitis, including *Fusobacterium nucleatum* and *Escherichia coli*, the latter producing gaseous metabolites in response to bile exposure. The role of these gaseous metabolites, particularly hydrogen, in either exacerbating or alleviating acute pancreatitis warrants further investigation through *in vitro* and *in vivo* studies involving larger patient cohorts across different stages of the disease.

## Data Availability

The datasets presented in this study can be found in online repositories. The names of the repository/repositories and accession number(s) can be found here: https://www.ncbi.nlm.nih.gov/, PRJNA1114082.
