# Supplementary figures and images for: Microbial dynamics of acute pancreatitis: integrating culture, sequencing, and bile impact on bacterial populations and gaseous metabolites
Source: Front Microbiol. 2025 Feb 12;16:1544124. doi: 10.3389/fmicb.2025.1544124 (PMC11860950; doi:10.3389/fmicb.2025.1544124)

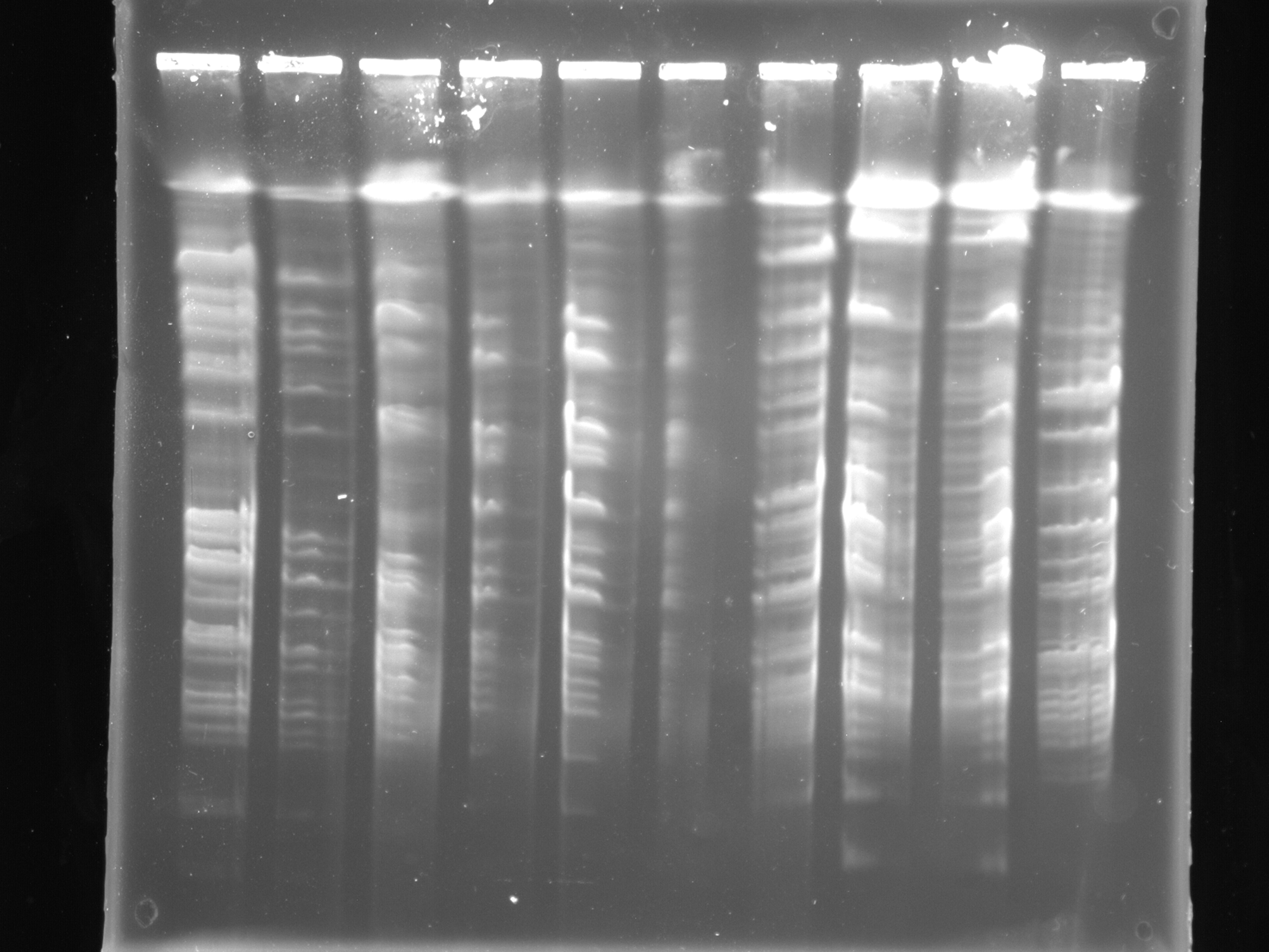

Supplement: Supplementary file 1 [file Image_1.tif]

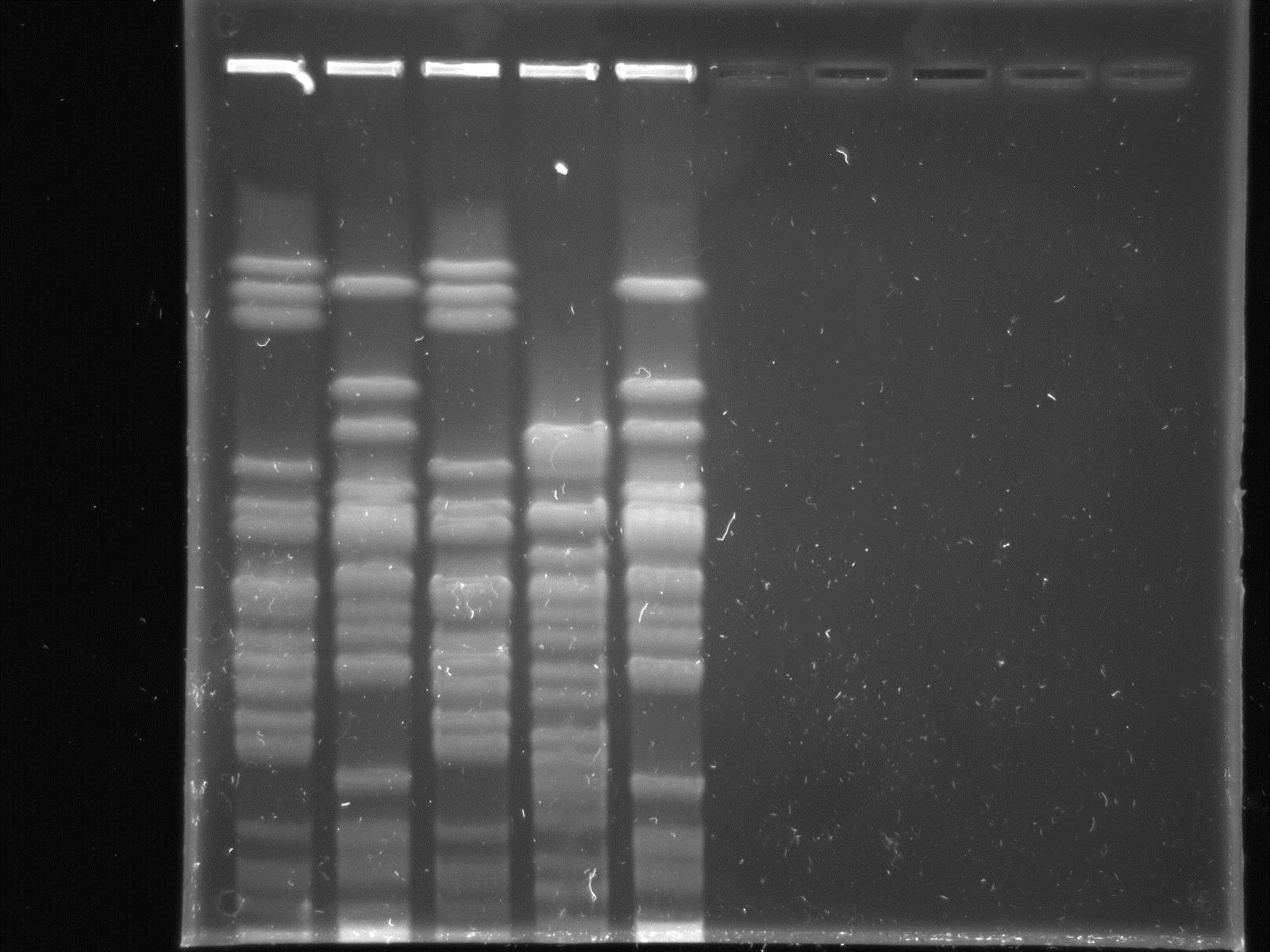

Supplement: Supplementary file 2 [file Image_2.tiff]
